# A randomised double-blind placebo-controlled clinical trial of oral hydroxyurea for transfusion-dependent β-thalassaemia

**DOI:** 10.1038/s41598-022-06774-8

**Published:** 2022-02-17

**Authors:** Nirmani Yasara, Nethmi Wickramarathne, Chamila Mettananda, Ishari Silva, Nizri Hameed, Kumari Attanayaka, Rexan Rodrigo, Nirmani Wickramasinghe, Lakshman Perera, Aresha Manamperi, Anuja Premawardhena, Sachith Mettananda

**Affiliations:** 1 Department of Paediatrics, Faculty of Medicine, University of Kelaniya, Thalagolla Road, Ragama, 11010 Sri Lanka; 2 Department of Pharmacology, Faculty of Medicine, University of Kelaniya, Ragama, Sri Lanka; 3 Adult and Adolescent Thalassaemia Centre, Colombo North Teaching Hospital, Ragama, Sri Lanka; 4 Molecular Medicine Unit, Faculty of Medicine, University of Kelaniya, Ragama, Sri Lanka; 5 Department of Medicine, Faculty of Medicine, University of Kelaniya, Ragama, Sri Lanka; 6 University Paediatrics Unit, Colombo North Teaching Hospital, Ragama, Sri Lanka

**Keywords:** Randomized controlled trials, Anaemia, Therapeutics

## Abstract

Hydroxyurea is an antimetabolite drug that induces fetal haemoglobin in sickle cell disease. However, its clinical usefulness in β-thalassaemia is unproven. We conducted a randomised, double-blind, placebo-controlled clinical trial to evaluate the efficacy and safety of hydroxyurea in transfusion-dependent β-thalassaemia. Sixty patients were assigned 1:1 to oral hydroxyurea 10–20 mg/kg/day or placebo for 6 months by stratified block randomisation. Hydroxyurea treatment did not alter the blood transfusion volume overall. However, a significantly higher proportion of patients on hydroxyurea showed increases in fetal haemoglobin percentage (89% vs. 59%; *p* < 0.05) and reductions in erythropoietic stress as measured by soluble transferrin receptor concentration (79% vs. 40%; *p* < 0.05). Based on fetal haemoglobin induction (> 1.5%), 44% of patients were identified as hydroxyurea-responders. Hydroxyurea-responders, required significantly lower blood volume (77 ± SD27ml/kg) compared to hydroxyurea-non-responders (108 ± SD24ml/kg; *p* < 0.01) and placebo-receivers (102 ± 28ml/kg; *p* < 0.05). Response to hydroxyurea was significantly higher in patients with HbE β-thalassaemia genotype (50% vs. 0%; *p* < 0.01) and *Xmn1* polymorphism of the γ-globin gene (67% vs. 27%; *p* < 0.05). We conclude that oral hydroxyurea increased fetal haemoglobin percentage and reduced erythropoietic stress of ineffective erythropoiesis in patients with transfusion-dependent β-thalassaemia. Hydroxyurea reduced the transfusion burden in approximately 40% of patients. Response to hydroxyurea was higher in patients with HbE β-thalassaemia genotype and *Xmn*1 polymorphism of the γ-globin gene.

## Introduction

β-Thalassaemia is a disorder of haemoglobin synthesis characterised by severe anaemia from late infancy^1^. Based on the requirement for red blood cell transfusions, β-thalassaemia is broadly divided into two groups: transfusion-dependent and non-transfusion-dependent β-thalassaemia^2^. Patients with transfusion-dependent β-thalassaemia that includes β-thalassaemia major and severe haemoglobin E (HbE) β-thalassaemia require regular 2–5 weekly blood transfusions for life^3^. Conversely, patients with non-transfusion-dependent β-thalassaemia, which encompasses β-thalassaemia intermedia and mild to moderate HbE β-thalassaemia require blood transfusions only occasionally^4^.

Allogenic haematopoietic stem cell transplantation is the only cure for β-thalassaemia at present^5^. However, it is available only to a minority of patients due to limitations in suitable donors and costs. Luspatercept, an activin IIB receptor ligand trap that inhibits ineffective erythropoiesis, has recently been approved to treat adult patients with β-thalassaemia. Nonetheless, it showed only a 33% reduction in the transfusion burden^6^. Several novel treatment modalities that include gene therapy and genome editing have shown promise in preclinical studies and clinical trials^7,8^. Again, none of these has progressed up to a level of standard patient care thus far. Therefore, most patients with transfusion-dependent β-thalassaemia only receive supportive treatment with regular blood transfusions for life^9^. Regular transfusions lead to iron overload resulting in irreversible organ damage and ultimate death of these patients in the fourth or fifth decade^10^. Thus, there is a great need for alternative therapies to reduce the blood transfusion burden of patients with transfusion-dependent β-thalassaemia.

Hydroxyurea is an antimetabolite S-phase-specific drug that reversibly inhibits ribonucleoside diphosphate reductase enzyme. It has shown to upregulate human γ-globin and fetal haemoglobin (HbF) synthesis in several in vitro and in vivo studies in patients with sickle cell disease^11,12^. Similarly, hydroxyurea induces HbF and reduces the blood transfusion requirement in some patients with non-transfusion-dependent β-thalassaemia^13^. In addition to its effect on HbF, hydroxyurea has been shown to improve haemorheology and inhibit ineffective erythropoiesis in patients with sickle cell disease and β-thalassaemia. At the cellular level, hydroxyurea alters epigenetics and proteomics to promote the synthesis of antioxidants, structural proteins, carbonic anhydrase and proteins required for protein repair, which may contribute to its beneficial effects on ineffective erythropoiesis^11^.

Despite being in use for non-transfusion dependent β-thalassaemia, due to the lack of well-designed clinical trials, the usefulness of hydroxyurea in transfusion-dependent β-thalassaemia is uncertain. The few published studies that evaluate the effects of hydroxyurea in this group of patients have provided conflicting results with variable response rates^14–18^. All these were observational studies with relatively small sample sizes. A meta-analysis of eleven observational studies reported that treatment with hydroxyurea results in a complete response rate of 26% in patients with transfusion-dependent β-thalassaemia^19^. However, two recent Cochrane reviews concluded that the available evidence from clinical trials is insufficient to show hydroxyurea is effective in patients with transfusion-dependent β-thalassaemia, and recommended well-designed clinical trials^19,20^. Here, in this clinical trial, we aim to evaluate the efficacy and safety of oral hydroxyurea in patients with transfusion-dependent β-thalassaemia.

## Results

**Figure 1:**
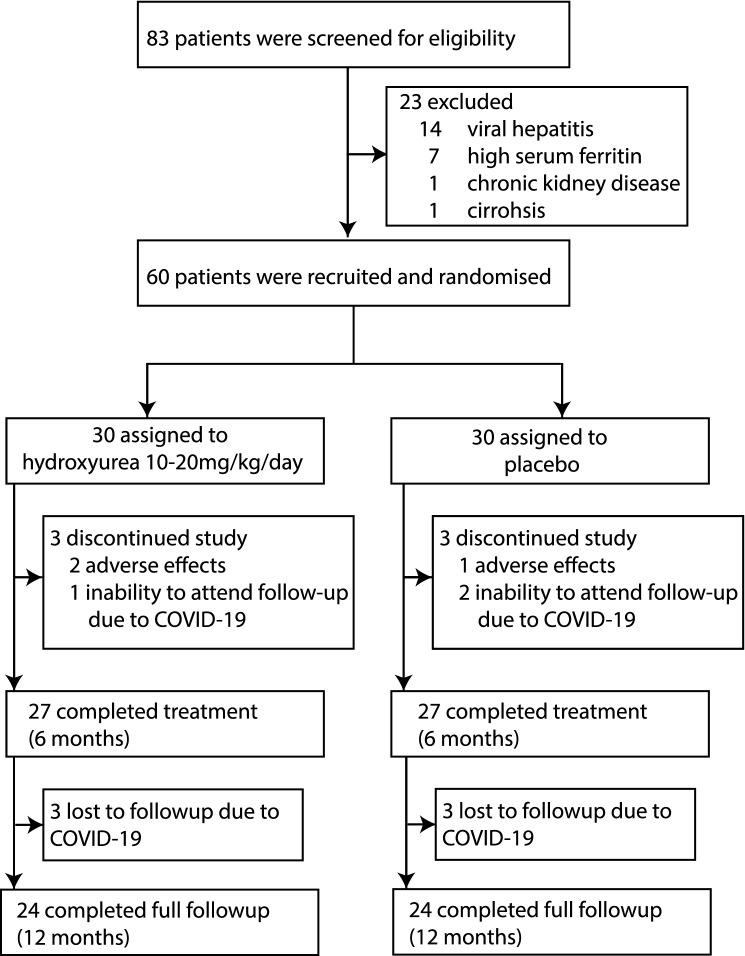
Trial profile showing participant flow during the trial.

A total of 60 patients with transfusion-dependent β-thalassaemia were enrolled into the study. Thirty participants each were randomly assigned to hydroxyurea and placebo arms (Fig. 1). A majority of participants (47 [78%]) had homozygous β-thalassaemia major while 13 (22%) had HbE β-thalassaemia. The socio-demographic, clinical, laboratory and genotype characteristics of hydroxyurea and placebo arms at enrolment were similar (Table 1).

**Table 1:** Baseline socio-demographic, clinical, laboratory and genotype characteristics of the intention to treat population.

Characteristic	Hydroxyurea arm (N = 30)	Placebo arm (N = 30)	*p*-value
Sex (Male: Female)	14:16	14:16	1.0
Age (years)	21.9 (± 7.9)	23.4 (± 7.9)	0.45
Ethnicity (Sinhala)	25 (83%)	29 (97%)	0.08
Monthly family income (< LKR 25,000)	20 (67%)	15 (50%)	0.19
Age at diagnosis (months)	17.5 (± 35.1)	13.0 (± 15.6)	0.53
β-thalassaemia type			
β-thalassaemia major	24 (80%)	23 (77%)	0.75
HbE β-thalassaemia	6 (20%)	7 (23%)	0.75
β-thalassaemia mutation^a^			
IVS1-5(G-C)/IVS1-5(G-C) (β^0^/β^0^)	12 (41%)	15 (52%)	0.61
IVS1-5(G-C)/IVS1-1(G-A) (β^0^/β^0^)	5 (17%)	5 (17%)	
IVS1-5(G-C)/CD16(-C) (β^0^/β^0^)	3 (10%)	1 (3%)	
CD16(-C)/CD16(-C) (β^0^/β^0^)	1 (3%)	0	
IVS1-1(G-A)/CD15(G-A) (β^0^/β^0^)	0	1 (3%)	
CD8/9(+ G)/CD8/9(+ G) (β^0^/β^0^)	1 (3%)	0	
IVS1-5(G-C)/CD26 (β^0^/β^E^)	6 (20%)	7 (24%)	
Blood transfusion history			
Age at first transfusion (months)	19.7 (± 36.2)	13.3 (± 15.7)	0.37
Number of transfusions during last 12 months	12.7 (± 2.7)	13.8 (± 3.8)	0.22
RBC units transfused during last 12 months	28.7 (± 8.8)	30.0 (± 9.6)	0.60
Organomegaly status			
Hepatomegaly	6 (20%)	6 (20%)	1.0
Splenomegaly	12 (40%)	6 (20%)	0.14
Splenectomised	5 (16.7%)	8 (26.7%)	0.34
Complications			
Short stature	7 (23%)	7 (23%)	1.00
Hypothyroidism	2 (7%)	3 (10%)	0.64
Hypoparathyroidism	7 (23%)	6 (20%)	0.75
Hypogonadism	7 (23%)	7 (23%)	1.00
Diabetes	3 (10%)	4 (13%)	0.68
Osteoporosis	0	1 (3%)	0.31
Laboratory characteristics at enrolment			
Haemoglobin	8.1 (± 0.9)	8.0 (± 1.0)	0.65
Mean corpuscular volume (fL)	77.0 (± 4.9)	77.6 (± 5.6)	0.63
Mean corpuscular haemoglobin (pg)	26.4 (± 2.1)	26.4 (± 1.6)	0.90
Mean corpuscular haemoglobin concentration (g/dL)	34.4 (± 1.4)	34.0 (± 1.9)	0.45
Red cell count (10^6^/mm^3^)	3.1 (± 0.3)	3.2 (± 0.9)	0.49
Haemoglobin A	88.4 (± 15.5)	87.0 (± 15.0)	0.73
Haemoglobin F	4.2 (± 6.8)	5.4 (± 6.5)	0.50
Haemoglobin E^b^	20.4 (± 9.9)	19.6 (± 8.6)	0.88
Haemoglobin A_2_	2.7 (± 0.4)	2.8 (± 0.4)	0.82
Serum ferritin (ng/mL)	1297 (± 803)	1372 (± 1030)	0.75
Alanine transaminase (u/L)	38.4 (± 31.2)	34.9 (± 27.5)	0.64
Serum creatinine (μmol/L)	59.8 (± 19.8)	65.9 (± 19.2)	0.25
Serum soluble transferrin receptor concentration (nmol/L)^c^	89.6 (± 22.8)	91.9 (± 24.7)	0.76

Data are n (%) or mean (± SD). *P*-values are from χ^2^ or independent sample t-test.

^a^Data from 57 patients.

^b^Data from HbE β-thalassaemia patients.

^c^Data from 40 patients; 24 hydroxyurea and 16 placebo.

Six participants (three from each arm) did not complete the treatment. Three of them discontinued the trial due to adverse events, while the other three discontinued due to their inability to attend the follow-up visits because of the COVID-19 pandemic. Further, six participants who completed the treatment and included in the analysis were lost to follow-up during the post-treatment period (Supplementary Table 1).

### Blood transfusion volume

The reduction in blood transfusion volume following hydroxyurea treatment was evaluated as the primary outcome measure. We did not observe a significant difference in the blood transfusion volume between hydroxyurea and placebo arms during the 6-month treatment period or post-treatment 6-months (Table 2).

**Table 2:** Blood transfusion volume (mL/kg) during the treatment and post-treatment follow-up periods.

	Hydroxyurea	Placebo	Difference in the mean (95% CI)	*p*-value
Treatment period	(N = 27)	(N = 27)		
First 3 months	41.9 (± 12.2)	45.7 (± 14.3)	− 3.8 (− 11.1 to 3.5)	0.30
Second 3 months	52.7 (± 20.5)	56.4 (± 16.1)	− 3.7 (− 13.8 to 6.3)	0.46
Entire 6 months	94.6 (± 29.8)	102.1 (± 28.0)	− 7.5 (− 23.3 to 8.3)	0.35
Post-treatment period	(N = 24)	(N = 24)		
First 3 months	46.8 (± 13.3)	47.2 (± 15.6)	− 0.4 (− 8.8 to 8.0)	0.93
Second 3 months	50.1 (± 19.4)	53.4 (± 16.7)	− 3.3 (− 13.8 to 7.2)	0.53
Entire 6 months	97.0 (± 29.0)	100.6 (± 27.3)	− 3.6 (− 20.0 to 12.7)	0.66

Data are mean (± SD).

### Change in fetal haemoglobin percentage

**Figure 2:**
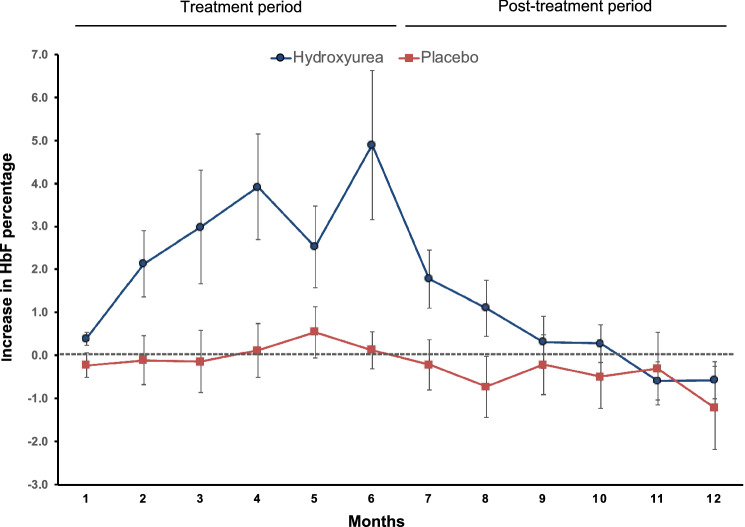
Line diagram showing the trends of rise in fetal haemoglobin percentage during treatment and post-treatment period. Mean increase of fetal haemoglobin from the baseline in hydroxyurea and placebo arms are shown. Error bars represent the standard error of mean. The mean and standard deviation values are given in Supplementary Table 2 (Figure created using Microsoft Excel version 16.57).

The induction of HbF was evaluated as a secondary outcome measure. A significantly higher proportion (89%) of participants in the hydroxyurea arm showed an increase in the HbF percentage during the treatment period compared to the placebo arm (59%) (Table 3 and Supplementary Tables 2 and 3). In the hydroxyurea arm, 12 (44%) showed an increase of HbF over 1.5% and 6 (22%) showed an increase of HbF over 5% (Supplementary Table 4). Similarly, the mean increase in HbF percentage and total HbF were significantly higher in the hydroxyurea arm compared to the placebo arm during the treatment period (Table 4). This difference disappeared during the post-treatment 6 months (Fig. 2).

**Table 3:** Improvements in the secondary outcome measures following treatment.

	Hydroxyurea (N = 27)	Placebo (N = 27)	Risk ratio (95% CI)	*p*-value
Increase in fetal haemoglobin percentage	24 (89%)	16 (59%)	5.5 (1.3 to 22.8)	0.013
> 1.5% increase in fetal haemoglobin percentage	12 (44%)	5 (18%)	3.5 (1.02 to 12.0)	0.04
> 5% increase in fetal haemoglobin percentage	6 (22%)	0	–	0.009
Decrease in serum soluble transferrin receptor concentration	19/24 (79%)	6/15 (40%)	5.7 (1.4 to 23.7)	0.013
> 10% decrease in serum soluble transferrin receptor concentration	14/24 (58%)	2/15 (13%)	9.1 (1.7 to 49.6)	0.005
> 25% decrease in serum soluble transferrin receptor concentration	8/24 (33%)	0/15	–	0.012

Data are n (%) or n/N (%).

**Table 4:** Increase in fetal haemoglobin percentage and total fetal haemoglobin from baseline during the treatment and post-treatment periods.

	Hydroxyurea	Placebo	Difference in the mean (95% CI)	*p*-value
**Increase in fetal haemoglobin percentage (%)**				
Treatment period	(N = 27)	(N = 27)		
First 3 months	2.10 (± 4.00)	− 0.28 (± 2.52)	2.38 (0.55 to 4.21)	0.012
Second 3 months	4.21 (± 6.89)	0.35 (± 2.29)	3.86 (1.05 to 6.66)	0.008
Entire 6 months	3.25 (± 5.29)	0.09 (± 2.04)	3.16 (0.97 to 5.35)	0.006
Post-treatment period	(N = 24)	(N = 24)		
First 3 months	1.10 (± 2.74)	− 0.26 (± 2.76)	1.36 (− 0.23 to 2.96)	0.09
Second 3 months	− 0.31 (± 1.68)	− 0.46 (± 3.68)	0.15 (− 1.59 to 1.88)	0.86
Entire 6 months	0.65 (± 2.50)	− 0.32 (± 3.06)	0.98 (− 0.64 to 2.60)	0.23
**Increase in total fetal haemoglobin (g/dL)**				
Treatment period	(N = 27)	(N = 27)		
First 3 months	0.15 (± 0.26)	− 0.01 (± 0.16)	0.15 (0.03 to 0.27)	0.011
Second 3 months	0.32 (± 0.50)	0.02 (± 0.16)	0.29 (0.09 to 0.50)	0.005
Entire 6 months	0.24 (± 0.37)	0.01 (± 0.13)	0.23 (0.07 to 0.38)	0.004
Post-treatment period	(N = 24)	(N = 24)		
First 3 months	0.09 (± 0.23)	− 0.02 (± 0.21)	0.12 (− 0.01 to 0.25)	0.06
Second 3 months	− 0.03 (0.13)	− 0.04 (0.24)	0.01 (− 0.10 to 0.13)	0.83
Entire 6 months	0.05 (0.21)	− 0.03 (0.22)	0.08 (− 0.04 to 0.21)	0.17

Data are mean (± SD).

### Change in erythropoietic stress levels of ineffective erythropoiesis

Erythropoietic stress of ineffective erythropoiesis was measured before and after treatment by assessing the serum soluble transferrin receptor (sTfR) concentration as a secondary outcome measure. A significantly higher proportion (79%) of patients in the hydroxyurea arm showed a decrease in serum sTfR concentration after treatment compared to the placebo arm (40%) (Table 3). In the hydroxyurea arm, 58% showed more than a 10% decrease, while 33% showed more than a 25% decrease in serum sTfR concentration compared to the pre-treatment levels (Supplementary Table 5). Similarly, the mean sTfR concentration in the hydroxyurea arm [72.2(SD ± 25.9)nmol/L, N = 24] was significantly lower than that of the placebo arm [94.4(SD ± 18.4)nmol/L, N = 15] at the end of the treatment period [difference in mean, − 22.1 (95%CI − 37.7 to − 6.5), *p* = 0.007].

### Hydroxyurea-responders and hydroxyurea-non-responders

Based on the increase in HbF percentage, we identified 12/27 (44%) patients who showed over 1.5% increase in HbF percentage as hydroxyurea-responders. Hydroxyurea-responders received significantly lower blood transfusion volume than the hydroxyurea-non-responders and placebo group throughout the treatment and post-treatment periods (Table 5). The mean blood transfusion volume received by hydroxyurea-responders was 24% lower than that received by patients in the placebo arm. Despite receiving lower blood volumes, the mean pre-transfusion haemoglobin levels of hydroxyurea-responders were similar to hydroxyurea-non-responders throughout treatment and post-treatment periods (Supplementary Table 6).

**Table 5:** Blood transfusion volume (mL/kg) received by hydroxyurea-responders, hydroxyurea- non-responders and placebo receivers.

	Hydroxyurea-responders		Hydroxyurea-non-responders		Difference in the mean transfusion volume (95% CI)	*p*-value
	Increase in HbF %	Transfusion volume (mL/kg)	Increase in HbF %	Transfusion volume (mL/kg)		
Treatment period	(N = 12)	(N = 12)	(N = 15)	(N = 15)		
First 3 months	4.58 (± 5.04)	36.1 (± 12.2)	0.11 (± 0.52)	46.6 (± 10.5)	− 10.4 (− 19.4 to − 1.4)	0.024
Second 3 months	9.00 (± 8.17)	41.3 (± 18.7)	0.37 (± 0.42)	61.8 (± 17.4)	− 20.5 (− 34.9 to − 6.2)	0.007
Entire 6 months	6.98 (± 6.20)	77.4 (± 27.9)	0.25 (± 0.33)	108.4 (± 24.0)	− 31.0 (− 51.6 to − 10.4)	0.005
Post-treatment period	(N = 9)	(N = 9)	(N = 15)	(N = 15)		
First 3 months	3.17 (± 3.63)	42.0 (± 9.7)	− 0.13 (± 0.58)	49.8 (± 14.5)	− 7.8 (− 19.1 to 3.5)	0.17
Second 3 months	− 0.87 (± 2.74)	35.6 (± 15.9)	− 0.05 (± 0.87)	58.8 (± 16.0)	− 23.2 (− 37.1 to − 9.2)	0.002
Entire 6 months	1.90 (± 3.79)	77.6 (± 24.3)	− 0.09 (± 0.70)	108.6 (± 25.7)	− 31.0 (− 53.0 to − 8.9)	0.008

	Hydroxyurea-responders		Placebo receivers		Difference in the mean transfusion volume (95% CI)	*p*-value
	Increase in HbF %	Transfusion volume (mL/kg)	Increase in HbF %	Transfusion volume (mL/kg)		
Treatment period	(N = 12)	(N = 12)	(N = 27)	(N = 27)		
First 3 months	4.58 (± 5.04)	36.1 (± 12.2)	− 0.27 (± 2.52)	45.7 (± 14.4)	− 9.6 (− 19.2 to 0.1)	0.052
Second 3 months	9.00 (± 8.17)	41.3 (± 18.7)	0.34 (± 2.28)	56.4 (± 16.1)	− 15.1 (− 27.0 to − 3.2)	0.014
Entire 6 months	6.98 (± 6.20)	77.4 (± 27.9)	0.08 (± 2.04)	102.1 (28.1)	− 24.7 (− 44.4 to − 5.0)	0.015
Post-treatment period	(N = 9)	(N = 9)	(N = 24)	(N = 24)		
First 3 months	3.17 (± 3.63)	42.0 (± 9.7)	− 0.26 (± 2.76)	47.2 (± 15.6)	− 5.2 (− 16.7 to 6.2)	0.35
Second 3 months	− 0.87 (± 2.74)	35.6 (± 15.9)	− 0.46 (± 3.68)	53.4 (± 16.7)	− 17.7 (− 30.9 to − 4.6)	0.010
Entire 6 months	1.90 (± 3.79)	77.6 (± 24.3)	− 0.32 (± 3.06)	100.6 (± 27.3)	− 23.0 (− 44.1 to − 1.8)	0.034

Data are mean (± SD).

### Predictors of response to hydroxyurea

Next, we examined the predictors of hydroxyurea response by comparing clinical and genetic factors of hydroxyurea-responders and non-responders as exploratory outcome measures (Table 6). Effects of organomegaly, α- and β-globin genotypes and the *Xmn1* polymorphism of the γ-globin gene on response to hydroxyurea treatment were evaluated. All patients (100%) with transfusion-dependent HbE β-thalassaemia who were in the treatment arm were hydroxyurea-responders. Conversely, only 28% of patients with non-HbE β-thalassaemia were hydroxyurea-responders. Also, a significantly higher proportion of patients with *Xmn1* polymorphism of the γ-globin gene were hydroxyurea-responders than patients without this polymorphism (67% vs. 27%; *p* = 0.038). In addition, the presence of splenomegaly was significantly associated with response to hydroxyurea (*p* = 0.004). We then examined for any association between the increase in HbF percentage and the decrease in sTfR following hydroxyurea treatment (Supplementary Fig. 1). We did not observe any significant correlation between these two variables [r = − 0.07 (95% CI: − 0.29 to 0.30), *p* = 0.72].

**Table 6:** Distribution of genetic modifiers among hydroxyurea-responders and non-responders.

	Hydroxyurea-responders (N = 12)	Hydroxyurea-non-responders (N = 15)	Risk ratio (95% CI)	*p* value
Male sex	5 (42%)	6 (40%)	1.1 (0.2 to 5.0)	0.93
Age (> 18 years)	7 (58%)	9 (60%)	0.9 (0.2 to 4.3)	0.93
Hepatomegaly	4 (33%)	1 (7%)	7.0 (0.6 to 73.9)	0.07
Splenomegaly	8 (67%)	2 (13%)	13 (1.9 to 87)	0.004
Splenectomy	2 (17%)	3 (20%)	0.8 (0.1 to 5.7)	0.82
HbE β-thalassaemia genotype	6 (50%)	0 (0%)	–	0.003
α–globin gene deletion	0 (0%)	1 (7%)	–	0.55
*Xmn1* polymorphism of the γ-globin gene	8 (67%)	4 (27%)	5.5 (1.04–28.2)	0.038

Data are n (%).

### Adverse events

Hydroxyurea was well tolerated, and only 7 (23%) in the hydroxyurea arm and 2 (7%) in the placebo arm experienced adverse events during the treatment period (Supplementary Table 7). There were no long-term adverse effects reported during the 1-year follow-up period. Headache was reported by two patients in the hydroxyurea arm and one in the placebo arm. One male patient in the hydroxyurea arm discontinued the trial due to persistent headache, and his symptoms improved after stopping the drug. Persistent thrombocytopenia along with leukopenia was observed in one male patient during hydroxyurea treatment. He discontinued treatment, and his haematological parameters normalised after stopping hydroxyurea. Two patients developed urinary tract infections while on hydroxyurea, and one patient developed hyperpigmentation. One patient in the hydroxyurea arm reported nausea, vomiting and abdominal pain; however, she was later confirmed to have peptic ulcer disease by upper gastrointestinal endoscopy. One patient in the placebo arm discontinued treatment due to an eczematous skin rash.

## Discussion

Despite being one of the earliest diseases to be genetically characterised, β-thalassaemia remains a disease without a cure in most patients^21^. This is particularly true for a large proportion of patients living in poor and underdeveloped countries in South and Southeast Asia^22^. Most current research on therapeutics of thalassaemia have resulted in high-cost, technically demanding epigenetic and genetic-based therapies which would not be affordable to many at need^7,23–26^. Hence it is important to identify low-cost, easily accessible adjunct therapies that can either cure or ameliorate β-thalassaemia.

In the current study, we evaluated the efficacy and safety of hydroxyurea in patients with transfusion-dependent β-thalassaemia. Our results show that hydroxyurea significantly upregulates the HbF synthesis and reduces erythropoietic stress of ineffective erythropoiesis of these patients. Over 40% of patients showed a reasonable response to hydroxyurea in blood transfusion volume, thus receiving 24% less blood transfusion volume than the placebo arm. All previous clinical studies that evaluated the effects of hydroxyurea on patients with transfusion dependent β-thalassaemia are either observational studies or clinical trials without control arms^19,20,27^. To our knowledge, this is the first randomised, double-blind placebo-controlled clinical trial evaluating the efficacy of hydroxyurea in transfusion-dependent β-thalassaemia.

Reducing the transfused blood volume in a subset of patients with β-thalassaemia by hydroxyurea has beneficial effects. Firstly, it will reduce the transfusion-related iron overload and organ dysfunction, which is the prime complication that leads to premature death in thalassaemia patients^28^. Although we did not observe a significant difference in serum ferritin between hydroxyurea responders and non-responders or placebo group during the study period, reduced transfusion requirement is expected to decrease the iron burden of these patients long-term. Secondly, it will minimise the risk of transfusion-transmitted infections. Thirdly, many countries across the globe struggle to keep up with the demand for blood products, specially during disaster situations like the COVID-19 pandemic^29,30^. Consequently, most patients with β-thalassaemia are inadequately transfused and suffer from chronic anaemia. Therefore, reducing the transfusion burden of β-thalassaemia will have beneficial effects on individual patients and the health care system in general.

Other encouraging features of hydroxyurea as an adjunct treatment for transfusion dependent β-thalassaemia are the vast experience of its use for other indications, availability and low cost. Hydroxyurea is an FDA approved drug that has been in use for cancers and sickle cell diseases for many decades^11^. It is widely available and is priced low. The cost for a one-year treatment of hydroxyurea in Sri Lanka is approximately US$30 compared to US$900 for one-year blood transfusions, US$170,000 for one-year treatment of luspatercept, US$25,000 for a haematopoietic stem cell transplantation and US$2,000,000 for gene therapy^31^. Hence it will be a treatment option available to all patients with β-thalassaemia, including those who live in poorer countries.

Another important finding of this study is the demonstration of a reduction in erythropoietic stress following hydroxyurea treatment. Erythropoietic stress in patients with β-thalassaemia is primarily due to ineffective erythropoiesis, which plays a significant role in disease pathogenesis^32–34^. Hence, reducing erythropoietic stress would be expected to ameliorate the symptoms and signs of β-thalassaemia and improve the quality of life. In our study, the reduction of serum sTfR concentration following hydroxyurea treatment poorly correlated with the increase in HbF percentage. This shows that the reduction of erythropoietic stress is possibly not mediated through the well-characterised γ-globin induction pathway of hydroxyurea but through an independent pathway that may be related to its cytotoxic effects. On the other hand, hydroxyurea-responders continue to require lower blood volumes even during the post-treatment period despite having decreased HbF levels. This suggests that hydroxyurea may benefit patients with transfusion-depended β-thalassaemia more by reducing the ineffective erythropoiesis than inducing HbF^35^. Nonetheless, it is clear that hydroxyurea could be beneficial through multiple pathways in patients with β-thalassaemia.

This study also identified genetic factors that predict a good response to hydroxyurea treatment. It revealed that haemoglobin E mutation and the *Xmn1* polymorphism of the γ-globin gene as significant predictors of response to hydroxyurea. Notably, all patients with HbE β-thalassaemia in the treatment arm responded to hydroxyurea. This is vital because HbE β-thalassaemia is one of the most common subtypes of β-thalassaemia worldwide. These findings are useful in deciding individualistic treatment based on pharmacogenomics for patients with β-thalassaemia in the future.

One important limitation of the study is the relatively small sample size for the subgroup analysis. Although our results showed a clear benefit in patients with HbE β-thalassaemia and among patients with *Xmn1* polymorphism, smaller sample size would have precluded us from identifying benefits in rarer β-thalassaemia genotypes. However, it is unclear whether a larger study would provide any additional evidence for the use of hydroxyurea in other β-thalassaemia genotypes.

Another limitation is that we could not evaluate the ability of hydroxyurea to maintain a safe but lower steady-state haemoglobin level of approximately 7 g/dL without transfusions. This was not feasible as it was considered unethical during the design of the study to withhold transfusions in patients when haemoglobin drops below 9 g/dL. However, the results of the current study will ethically justify future studies to discontinue regular transfusions and carefully monitor haemoglobin levels during hydroxyurea treatment. If blood transfusions could be withheld until haemoglobin drops to 7–8 g/dL, it is likely that a higher response to hydroxyurea would be observed as active erythropoiesis is a prerequisite for the action of hydroxyurea.

In conclusion, we have presented the results of the first randomised, double-blind placebo-controlled clinical trial that evaluated the efficacy and safety of hydroxyurea in patients with transfusion-dependent β-thalassaemia. We found that hydroxyurea significantly upregulates fetal haemoglobin and reduces erythropoietic stress of ineffective erythropoiesis in these patients. Hydroxyurea reduced the transfusion burden in approximately 40% of patients, and the response to hydroxyurea was higher among patients with HbE β-thalassaemia genotype and *Xmn1* polymorphism of the γ-globin gene. There were no permanent adverse effects reported. Based on the results, it is justifiable to recommend a therapeutic trial of hydroxyurea as an adjunct therapy for transfusion-dependent β-thalassaemia at least in patients with favourable genetic factors.

## Methods

### Study design and participants

We conducted a randomised, double-blind, placebo-controlled clinical trial at the Adult and Adolescent Thalassaemia Centre of Colombo North Teaching Hospital, Ragama, Sri Lanka, from January 2019 to August 2021. Patients with transfusion-dependent β-thalassaemia aged over 12 years and required more than eight blood transfusions during the proceeding one year were eligible to participate in the study^36^. Patients with sickle β-thalassaemia; coexisting chronic kidney disease, cirrhosis or viral hepatitis; contraindications for hydroxyurea (hypersensitivity, bone marrow failure, pregnancy and lactation); very high serum ferritin (> 5000 ng/mL); leukopenia (< 4000/μL); and thrombocytopenia (< 150,000/μL) were excluded^36^. Eligible patients were recruited after obtaining informed written consent.

### Sample size

A recent meta-analysis of previous observational studies reported that hydroxyurea is associated with a 26% complete response rate among patients with transfusion dependent β-thalassaemia^19^. The sample size was calculated based on this study for a 1:1 enrolment ratio, a type I error (alpha) of 0.05, a type II error (beta) of 0.2 and a power of 80%. The minimal sample size required was 25 per arm.

### Randomisation and masking

After recruitment, participants were assigned to intervention or control arms using a stratified block randomisation method using an online randomisation tool. Participants were stratified by gender and randomised in fixed block sizes of four into intervention or control arms in a 1:1 ratio. The intervention arm received oral hydroxyurea, whereas the control arm received a placebo capsule which is identical in size, shape and colour to the hydroxyurea capsule without its active ingredient. Hydroxyurea or placebo capsules were packed in sealed envelopes labelled with the trial number at a third-party location and handed over to the investigators^36^. Participants, data collectors, outcome adjudicators and data analysts were blinded regarding the treatment until the final data analysis^36^.

### Procedures

**Figure 3:**
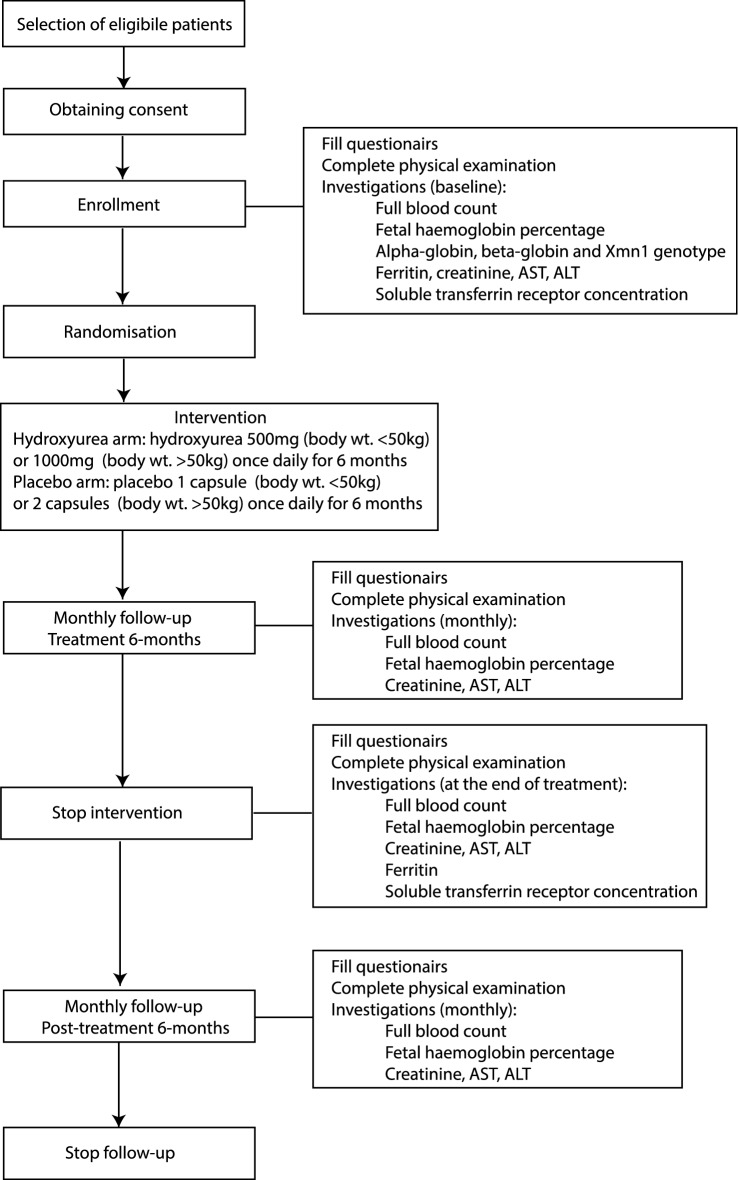
Flow diagram showing study protocol. AST- aspartate aminotransferase, ALT- alanine aminotransferase.

Participants' basic socio-demographic and medical data were gathered at enrolment, and the following laboratory investigations were done; full blood count, HbF percentage, β-globin and α-globin genotypes, *Xmn1* genotype, serum ferritin, serum creatinine, aspartate aminotransferase and alanine aminotransferase (Fig. 3). Participants in the intervention arm were given oral hydroxyurea (manufactured by Cadila Healthcare Ltd, India) 10–20 mg/kg daily for 6 months. Patients who weighed less than 50 kg were given one 500 mg capsule of hydroxyurea, and those who weighed 50–100 kg were given two 500 mg capsules once daily. Patients in the control arm were given the same number of capsules per body weight of a placebo capsule (manufactured by State Pharmaceutical Manufacturing Cooperation, Sri Lanka) for 6 months^36^.

All other standard treatments were continued during the trial, and blood transfusions were given when haemoglobin dropped below 9 g/dL. Study participants were reviewed at least monthly during the 6-month treatment period and for further 6 months after stopping treatment (post-treatment period)^36^. Data on blood transfusion volume, haemoglobin and HbF percentage were obtained monthly during each follow-up visit, and patients were screened for clinical and laboratory evidence of adverse effects of treatment. sTfR concentration was measured at the beginning and end of the 6-month treatment period in a limited number of participants due to limitations of the assay availability. The clinician caring for the patients was blinded with regards to the treatment allocation, HbF percentage and the sTfR concentration of study participants.

### Outcomes

Reduction in blood transfusion volume during the treatment period was the primary outcome measure. The secondary outcome measures were increases in HbF percentage, reductions in erythropoietic stress of ineffective erythropoiesis as measured by a decrease in serum sTfR, and adverse events. The increase in HbF percentage was calculated by comparing the average HbF levels during the 6-month treatment period with the baseline HbF value. The decrease in serum sTfR was calculated comparing the measurements taken at the beginning and end of the 6-month treatment period. Adverse events during treatment were assessed by direct questioning about the known side effects of hydroxyurea and by performing a full blood count at each follow-up visit to evaluate for haematological toxicity. Effects of α- and β-globin genotypes and the *Xmn1* polymorphism of the γ-globin gene on response to hydroxyurea treatment were evaluated as exploratory outcome measures.

### Statistical analysis

Blood transfusion volume, increase in HbF percentage, and the percentage reduction of serum sTfR concentration were compared between hydroxyurea and placebo groups by per-protocol analysis. Adverse events in hydroxyurea and placebo groups were compared by intension-to-treat analysis. Data were analysed using IBM SPSS statistic version 25.0.

### Ethical considerations

All work related to this research was carried out in accordance with the Declaration of Helsinki. Ethical approval was obtained from the Ethics Committee of the Faculty of Medicine, University of Kelaniya, Sri Lanka (P/116/05/2018). All participants were recruited after obtaining informed written consent. The complete study protocol was published in *BMJ Open* (Link: https://bmjopen.bmj.com/content/10/10/e041958)^36^, and the trial was registered in the Sri Lanka Clinical Trials Registry as SLCTR/2018/024 on 01/08/2018.

## Supplementary Information

Supplementary Information.

## Data Availability

Individual participant data will not be shared due to ethical restrictions. The anonymised datasets generated and analysed during the current study are available from the corresponding author on reasonable request.
