# Supplementary material for: A randomised double-blind placebo-controlled clinical trial of oral hydroxyurea for transfusion-dependent β-thalassaemia
Source: Sci Rep. 2022 Feb 17;12:2752. doi: 10.1038/s41598-022-06774-8 (PMC8854735; doi:10.1038/s41598-022-06774-8)
Supplement: Supplementary file 1 — Supplementary Information. [file 41598_2022_6774_MOESM1_ESM.docx]

**Supplementary information**

**Manuscript title:** A randomised double-blind placebo-controlled clinical trial of oral hydroxyurea for transfusion-dependent β-thalassaemia

**Supplementary Table 1. Information of study dropouts**

| **Study number** | **Treatment arm** | **Sex** | **Age (years)** | **Type of thalassaemia** | **Timepoint of dropout (days)** | **Reason** |
| --- | --- | --- | --- | --- | --- | --- |
| M/5 | Hydroxyurea | Male | 27 | β-thalassaemia major | 29 | Headache |
| M/7 | Hydroxyurea | Male | 27 | β-thalassaemia major | 49 | Pancytopenia |
| M/23 | Placebo | Male | 24 | β-thalassaemia major | 7 | Skin rash |
| M/28 | Hydroxyurea | Male | 12 | β-thalassaemia major | 81 | Due to COVID-19 pandemic |
| F/132 | Placebo | Female | 38 | HbE β-thalassaemia | 60 | Due to COVID-19 pandemic |
| F/120 | Placebo | Female | 16 | HbE β-thalassaemia | 70 | Due to COVID-19 pandemic |
| F/121 | Hydroxyurea | Female | 45 | HbE β-thalassaemia | > 200 | Due to COVID-19 pandemic |
| F/131 | Hydroxyurea | Female | 14 | HbE β-thalassaemia | > 200 | Due to COVID-19 pandemic |
| F/133 | Hydroxyurea | Female | 35 | HbE β-thalassaemia | > 200 | Due to COVID-19 pandemic |
| M/26 | Placebo | Male | 12 | HbE β-thalassaemia | > 200 | Due to COVID-19 pandemic |
| M/27 | Placebo | Male | 42 | HbE β-thalassaemia | > 200 | Due to COVID-19 pandemic |
| F/128 | Placebo | Female | 18 | HbE β-thalassaemia | > 200 | Due to COVID-19 pandemic |

**Supplementary Table 2. Increase in the fetal haemoglobin percentage in comparison to the baseline value during treatment and post treatment periods**

|  | **Hydroxyurea** | **Placebo** | **Difference in the mean (95% CI)** | **p-value** |
| --- | --- | --- | --- | --- |
| Treatment period | (N=27) | (N=27) |  |  |
| 1^st^ month | 0.39 (±0.68) | -0.22 (±1.26) | 0.61 (-0.03 to 1.26) | 0.06 |
| 2^nd^ month | 2.13 (±3.53) | -0.10 (±2.67) | 2.23 (0.31 to 4.16) | 0.02 |
| 3^rd^ month | 2.98 (±6.33) | -0.13 (±0.72) | 3.12 (-0.31 to 6.56) | 0.07 |
| 4^th^ month | 3.92 (±6.27) | 0.12 (±2.86) | 3.80 (0.81 to 6.78) | 0.01 |
| 5^th^ month | 2.52 (±4.56) | 0.54 (±2.73) | 1.98 (-0.33 to 4.30) | 0.09 |
| 6^th^ month | 4.89 (±8.49) | 0.12 (±2.24) | 4.76 (1.36 to 8.17) | 0.007 |
|  |  |  |  |  |
| Post-treatment period | (N=24) | (N=24) |  |  |
| 7^th^ month | 1.78 (±3.31) | -0.21 (±2.68) | 1.99 (0.16 to 3.82) | 0.03 |
| 8^th^ month | 1.10 (±3.05) | -0.72 (±3.08) | 1.82 (-0.11 to 3.77) | 0.06 |
| 9^th^ month | 0.31 (±2.69) | -0.21 (±3.25) | 0.52 (-1.34 to 2.39) | 0.57 |
| 10^th^ month | 0.28 (±1.90) | -0.49 (±3.20) | 0.77 (-0.96 to 2.50) | 0.37 |
| 11^th^ month | -0.58 (±2.04) | -0.30 (±3.67) | -0.28 (-2.16 to 1.59) | 0.76 |
| 12^th^ month | -0.56 (±1.83) | -1.21 (±4.10) | 0.64 (-1.50 to 2.79) | 0.54 |

Data are mean (±SD).

**Supplementary Table 3. Increase in the total fetal haemoglobin (g/dL) in comparison to the baseline value during treatment and post treatment periods**

|  | **Hydroxyurea** | **Placebo** | **Difference in the mean (95% CI)** | **p-value** |
| --- | --- | --- | --- | --- |
| Treatment period | (N=27) | (N=27) |  |  |
| 1^st^ month | 0.03 (±0.05) | 0.00 (±0.09) | 0.03 (-0.01 to 0.08) | 0.20 |
| 2^nd^ month | 0.16 (±0.23) | -0.00 (±0.17) | 0.16 (0.03 to 0.29) | 0.01 |
| 3^rd^ month | 0.21 (±0.41) | 0.01 (±0.23) | 0.19 (-0.03 to 0.43) | 0.10 |
| 4^th^ month | 0.32 (±0.52) | 0.01 (±0.20) | 0.30 (0.06 to 0.55) | 0.01 |
| 5^th^ month | 0.21 (±0.39) | 0.04 (±0.19) | 0.16 (-0.02 to 0.35) | 0.86 |
| 6^th^ month | 0.34 (±0.52) | 0.00 (±0.15) | 0.33 (0.11 to 0.55) | 0.003 |
|  |  |  |  |  |
| Post-treatment period | (N=24) | (N=24) |  |  |
| 7^th^ month | 0.14 (±0.26) | -0.02 (±0.21) | 0.17 (0.02 to 0.31) | 0.022 |
| 8^th^ month | 0.10 (±0.27) | -0.05 (±0.21) | 0.15 (-0.01 to 0.32) | 0.051 |
| 9^th^ month | 0.02 (±0.22) | -0.02 (±0.25) | 0.04 (-0.09 to 0.19) | 0.50 |
| 10^th^ month | 0.02 (±0.16) | -0.02 (±0.24) | 0.05 (-0.08 to 0.19) | 0.43 |
| 11^th^ month | -0.05 (±0.17) | -0.02 (±0.25) | -0.02 (-0.16 to 0.11) | 0.71 |
| 12^th^ month | -0.05 (±0.14) | -0.08 (+0.27) | 0.03 (-0.12 to 0.18) | 0.68 |

Data are mean (±SD).

**Supplementary Table 4. Increase in the fetal haemoglobin percentage during treatment period in comparison to the baseline value**

| **Increase in the HbF percentage** | **Hydroxyurea arm (N=27)** | **Placebo arm**  **(N=27)** |
| --- | --- | --- |
| >=10.0 | 2 (7.4) | 0 |
| 5.00-9.99 | 4 (14.8%) | 0 |
| 3.00-4.99 | 2 (7.4%) | 1 (3.7%) |
| 1.50-2.99 | 4 (14.8%) | 4 (14.8%) |
| 0.01-1.49 | 12 (44.4%) | 11 (40.7%) |
| No increase | 3 (11.1%) | 11 (40.7%) |

Data are n (%)

**Supplementary Table 5. Percentage decrease in serum soluble transferrin receptor concentration from baseline following treatment**

| **Percentage decrease in serum soluble transferrin receptor concentration** | **Hydroxyurea arm (N=24)** | **Placebo arm**  **(N=15)** |
| --- | --- | --- |
| >=50.0 | 2 (8%) | 0 |
| 25.00-49.99 | 6 (25%) | 0 |
| 10.00-24.99 | 6 (25%) | 2 (13%) |
| 5.00-9.99 | 3 (13%) | 0 |
| 0.01-4.99 | 2 (8%) | 4 (26%) |
| No decrease | 5 (21%) | 9 (60%) |

Data are n (%)

**Supplementary Table 6. Pre-transfusion haemoglobin level of hydroxyurea-responders, hydroxyurea-non-responders and placebo receivers**

|  | **Hydroxyurea-responders** | **Hydroxyurea-non-responders** | **Difference in the mean (95% CI)** | **p-value** |
| --- | --- | --- | --- | --- |
| Treatment period | (N=12) | (N=15) |  |  |
| First 3 months | 8.02 (±0.87) | 8.44 (±0.48) | -0.41 (-0.96 to 0.12) | 0.12 |
| Second 3 months | 8.11 (±1.20) | 8.36 (±0.71) | -0.24 (-1.01 to 0.52) | 0.51 |
| Entire 6 months | 8.07 (±0.99) | 8.39 (±0.57) | -0.32 (-0.95 to 0.30) | 0.29 |
|  |  |  |  |  |
| Post-treatment period | (N=9) | (N=15) |  |  |
| First 3 months | 8.53 (±0.55) | 8.73 (±0.62) | - 0.19 (-0.71 to 0.33) | 0.45 |
| Second 3 months | 8.51 (±0.71) | 8.33 (±0.85) | 0.18 (-0.55 to 0.92) | 0.61 |
| Entire 6 months | 8.56 (±0.56) | 8.52 (±0.65) | 0.04 (-0.50 to 0.58) | 0.88 |
|  |  |  |  |  |
|  | **Hydroxyurea-responders** | **Placebo receivers** | **Difference in the mean (95% CI)** | **p-value** |
| Treatment period | (N=12) | (N=27) |  |  |
| First 3 months | 8.02 (±0.87) | 8.19 (±0.92) | - 0.16 (-0.80 to 0.47) | 0.60 |
| Second 3 months | 8.11 (±1.20) | 7.96 (±0.97) | 0.15 (-0.58 to 0.89) | 0.67 |
| Entire 6 months | 8.07 (±0.99) | 8.07 (±0.88) | -0.003 (-0.65 to 0.64) | 0.99 |
|  |  |  |  |  |
| Post-treatment period | (N=9) | (N=24) |  |  |
| First 3 months | 8.53 (±0.55) | 8.23 (±0.76) | 0.30 (-0.26 to 0.87) | 0.28 |
| Second 3 months | 8.51 (±0.71) | 8.09 (±0.95) | 0.42 (-0.33 to 1.17) | 0.26 |
| Entire 6 months | 8.56 (±0.56) | 8.16 (±0.76) | 0.40 (-0.16 to 0.98) | 0.15 |

Data are mean (±SD).

**Supplementary Table 7. Adverse events reported during treatment**

| **Adverse event** | **Hydroxyurea (N=30)** | **Placebo**  **(N=30)** |
| --- | --- | --- |
| Headache | 2 (6.6%) | 1 (3.3%) |
| Thrombocytopenia (Platelet count <150x10^9^/L) | 1 (3.3%) | 0 |
| Leukopenia (White cell count <4x10^9^/L) | 1 (3.3%) | 0 |
| Hyperpigmentation | 1 (3.3%) | 0 |
| Urinary tract infection | 2 (6.6%) | 0 |
| Nausea | 1 (3.3%) | 0 |
| Vomiting | 1 (3.3%) | 0 |
| Abdominal pain | 1 (3.3%) | 0 |
| Skin rash (eczematous) | 0 | 1 (3.3%) |

Data are n (%)

.

**Supplementary Figure 1. Scatter diagram showing the correlation between percentage decrease of soluble transferrin receptor concentration (sTfR) and increase in fetal haemoglobin (HbF) percentage.**
